# Acute Intracranial Subdural Hematoma Masquerading as a Postpartum Headache: A Case Report

**DOI:** 10.5811/cpcem.2023.1.59331

**Published:** 2023-02-09

**Authors:** Julie M. Tondt, Francis L. Counselman, Michael J. Bono

**Affiliations:** * Penn State Health Milton S. Hershey Medical Center, Department of Emergency Medicine, Hershey, Pennsylvania; † Eastern Virginia Medical School, Department of Emergency Medicine, Norfolk, Virginia; ‡ Emergency Physicians of Tidewater, Norfolk, Virginia

**Keywords:** case report, intracranial subdural hematoma, postpartum, epidural anesthesia, headache

## Abstract

**Introduction:**

An acute subdural hematoma is a collection of blood in the space between the dural and arachnoid membranes overlying the brain. Head trauma is the most common cause. Less frequently, low cerebrospinal fluid pressure, due to a spontaneous or iatrogenic cerebrospinal fluid leak can result in a subdural hematoma.

**Case Report:**

We discuss the case of a 26-year-old woman who presented with a frontal headache following epidural anesthesia for vaginal delivery. The differential diagnosis included spinal headache, postpartum hypercoagulability, dural sinus thrombosis, and intracranial hemorrhage or mass. Her vital signs and physical examination were normal. A computed tomography of the brain revealed an acute subdural hematoma along the left frontal cerebral hemisphere, without midline shift or mass effect. A blood patch was placed with complete resolution of her symptoms.

**Conclusion:**

This case illustrates an unusual case of an acute subdural hematoma in the postpartum period following epidural anesthesia for labor pain management. It was thought to be caused by intracranial hypotension following epidural anesthesia and a cerebrospinal fluid leak.

## INTRODUCTION

Over the past several decades in the United States (US), epidural anesthesia has become the most popular pain management option for women during labor and delivery.^1^,^2^ According to a 2011 study by the US Centers for Disease Control and Prevention, 61% of women in the US who vaginally delivered received epidural or spinal anesthesia across 27 states in 2008.^3^

Postdural puncture headache (PDPH) is a recognized complication following epidural anesthesia.^1^,^2^,^4^ According to the International Headache Society, a PDPH occurs within five days of a lumbar puncture due to leaking of cerebral spinal fluid (CSF), it is often postural in nature, and may spontaneously resolve in two weeks with conservative measures or with an epidural blood patch.^2^,^5^ First described by Dr. August Bier in 1869, PDPH is thought to occur due to unintentional dural puncture during epidural anesthesia, leaking of CSF, and either subsequent strain on intracranial structures or, alternatively, vasodilation resulting in a headache.^2^,^6^–^10^ According to a 2014 article by Gurudatt, the rate of unintentional dural puncture (UDP) during epidural anesthesia is described as a range 0.19–3.6%, with 60–80% of patients who experience an UDP going on to have a PDPH.^11^

Headaches in general are common in the postpartum period, with a 2005 prospective cohort study by Goldszmidt et al reporting an incidence of 39%.^12^ An uncommon yet neurologically important cause of postpartum headache following epidural anesthesia is an intracranial subdural hematoma (SDH). Similarly to PDPH, an intracranial SDH is thought to occur due to unintentional dural puncture during epidural anesthesia, leaking of CSF, and subsequent strain on intracranial neurovascular structures, specifically the bridging veins, leading to intracranial bleeding.^4^,^6^,^7^,^13^ Although the true incidence has not been reported, from our review of the literature, an often cited retrospective study by Scott and Hibbard reported an incidence of one SDH following unintentional dural puncture during epidural anesthesia for obstetrical patients out of approximately 505,000 cases in the United Kingdom between 1982–1986.^14^

We present the case of a 26-year-old woman who presented to the emergency department (ED) with an unusual presentation of an acute intracranial SDH masquerading as a postpartum headache following epidural anesthesia.

## CASE REPORT

A 26-year-old gravida 1, para 1 female presented to the ED reporting a frontal headache for the prior three days. The patient had received epidural anesthesia for an uncomplicated spontaneous vaginal delivery five days earlier and was discharged to home after two days. The patient reported that since being home she had been having a headache described as a pressure feeling. A friend recommended that she lie flat, which did improve her headache, but she was concerned because the headache returned every time she sat or stood upright. She denied fever, chills, nausea, vomiting, extremity numbness or weakness, chest pain, shortness of breath, or other associated symptoms. Past medical history was significant only for asthma. The patient was on no medications and denied alcohol use or cigarette smoking.

Physical exam revealed a young woman in no obvious distress. Vital signs revealed a pulse of 80 beats per minute, respiratory rate of 18 breaths per minute, blood pressure of 127/91 millimeters of mercury, temperature of 98° Fahrenheit (36.7° Celsius), and 96% oxygen saturation on room air. Examination of the head, eyes, ears, nose, and throat exam was normal. The heart exam was normal, and auscultation of the lungs revealed clear, bilateral breath sounds. The abdomen was soft, nontender, and without guarding or rebound. Examination of the back revealed no evidence of a CSF leak. On neurologic exam, she was awake, alert, and oriented to person, place, time, and situation. Cranial nerves II–XII were intact, the patient had 5/5 motor strength in all four extremities, and a normal gait. Sensation was intact to light touch throughout, and there was no upper extremity pronator drift.

*CPC-EM Capsule*

What do we already know about this clinical entity?

*Acute intracranial subdural hematoma is an uncommon yet cannot miss neurologic emergency in the postpartum patient presenting with a headache.*

What makes this presentation of disease reportable?

*An acute intracranial subdural hematoma was found in a postpartum patient presenting with a headache after receiving epidural anesthesia during labor.*

What is the major learning point?

*Acute intracranial subdural hematoma should be included in the differential diagnosis for postpartum patients reporting a headache after receiving epidural anesthesia.*

How might this improve emergency medicine practice?

*Consideration of acute intracranial subdural hematoma as a potential cause of headache in the postpartum patient can facilitate a timely diagnosis.*

An intravenous line was established. Laboratory studies were sent for a complete blood count (CBC), basic metabolic profile (BMP), liver function studies (LFTs), and a urinalysis. A non-contrast computed tomography (CT) of the head was also ordered. The CBC was only remarkable for a mild leukocytosis. The BMP and urinalysis were normal. The LFTs were remarkable for a mildly elevated alkaline phosphatase. The CT of the head (Image) revealed “an acute subdural hematoma along the left frontal cerebral hemispheric convexity, measuring 7 millimeters (mm) in thickness and 9 centimeters (cm) in the anterior posterior dimension. There was no midline shift or mass effect.”

Neurosurgery was not available at the hospital, so the emergency physician called the local tertiary care referral hospital and consulted neurosurgery. The neurosurgeon recommended coagulation studies, type and screen, testing for coronavirus disease 2019 (COVID-19), neuro checks every hour, bed rest, and levetiracetam 500 milligrams intravenously every 12 hours. Coagulation studies were normal, and the COVID-19 test was negative. Review of the anesthesia records revealed the epidural anesthesia had been placed with the patient in the sitting position at the third and fourth lumbar level. A 17-gauge epidural needle had been used and required only a single attempt. No complications were described.

The patient remained in stable condition and was transferred to the tertiary care hospital without incident. Her neurologic exam remained unchanged, and she arrived with a mild headache, no new complaints, and was overall in no acute distress. Neurosurgery ordered a computed tomography angiography (CTA) of the head and neck to evaluate for an etiology of the SDH. The study revealed “no explanation for the subdural hemorrhage. No aneurysm or arteriovenous malformation. Patent intracranial superficial and deep venous system. Patent intracranial and extracranial cerebral arteries without evidence of significant stenosis.” The patient was admitted to the neurology intensive care unit for close observation. Additional studies included magnetic resonance imaging (MRI) of the cervical, lumbar, and thoracic spines without contrast; all were unremarkable. An MRI head with and without contrast revealed “a left frontal convexity SDH measuring up to 7 mm in maximum thickness. No other hemorrhage identified.”

The patient remained in stable condition and was transferred to the floor the next day. Anesthesia placed an epidural blood patch, which resulted in significant improvement in her symptoms. She was discharged home the next day, pain free and with a normal neurologic exam.

## DISCUSSION

The broad differential diagnosis of postpartum headache may lead to a delay in the identification of SDH, as presenting symptoms often overlap with other processes, such as PDPH.^4^,^7^ In a 2010 literature review of 35 cases, Anorim et al reported that common risk factors for SDH following epidural anesthesia included pregnancy, multiple attempts, anticoagulants, intracranial vascular abnormalities, and cerebral atrophy, while noting that 15 (43%) of the cases had no reported risk factors.^6^ Our patient had no known past medical history and an otherwise uncomplicated pregnancy course.

Computed tomography is the initial imaging modality of choice to evaluate for SDH after epidural anesthesia in patients with a high clinical suspicion.^4^,^15^ Other imaging modalities, such as MRI or CTA, may be considered for further characterization of SDH. as well as evaluation of potential alternative causes aside from unintentional dural puncture, as with our case.^4^,^6^,^15^

Treatment recommendations for intracranial SDH following epidural anesthesia are based on patient presentation and clinical findings, and range from symptomatic treatment to emergent neurosurgical intervention.^4^,^6^–^7^,^13^,^15^ In a 2014 literature review, Cuypers et al reported that of 34 women who had an intracranial SDH following epidural anesthesia for vaginal or cesarean delivery, 50% needed urgent surgical treatment, 44% received conservative management, and a total of 88% of patients were reported to have complete recovery, while the remaining 12% either had permanent neurologic deficit or died as a result of the hematoma.^15^ Similar to our patient presentation, Vien et al presented a case of a 27-year-old nulliparous female patient with no known past medical history who was found to have subdural hygromas and a SDH following epidural anesthesia and had resolution of symptoms after a blood patch.^13^ Comparatively, Kale et al presented a case of a 34-year-old primigravida female with no known past medical history who reported a headache after epidural anesthesia for labor, subsequently developed focal neurological deficits, was found to have bilateral acute SDH necessitating a burr hole, and was reported to have eventual resolution of symptoms.^4^

Cuypers et al reported that of 34 women who had an intracranial SDH following epidural anesthesia for vaginal or cesarean delivery, 31 (91%) initially presented with PDPH, of whom 84% went on to have continued non-postural headache, and overall 71% were reported to have focal neurologic deficits.^15^ Emergency physicians should consider further evaluation for SDH in postpartum patients following epidural anesthesia who present with symptoms such as headaches that are persistent, no longer postural in nature, only temporarily relieved by conservative measures, and/or associated with focal neurologic deficits.^6^–^7^,^13^,^15^

## CONCLUSION

Acute intracranial subdural hematoma is an uncommon neurologic emergency that emergency physicians should consider in the differential diagnosis of postpartum patients presenting with a headache and history of epidural or spinal anesthesia. A thorough past medical history including obstetrical history, especially identifying those who received epidural anesthesia, is important to obtain from the postpartum patient presenting to the ED.

## Figures and Tables

**Image:**
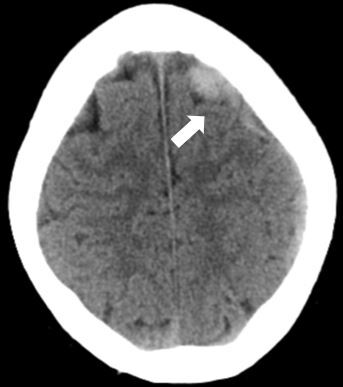
Transverse section of a computed tomography image of the head showing an acute subdural hematoma along the left frontal cerebral hemispheric convexity (arrow).
